# 胸壁骨性重建的研究进展

**DOI:** 10.3779/j.issn.1009-3419.2018.04.07

**Published:** 2018-04-20

**Authors:** 代星 钟, 磊 王, 小飞 李, 立军 黄

**Affiliations:** 710038 西安，空军军医大学唐都医院胸腔外科 Department of Thoracic Surgery, Tangdu Hospital, Fourth Military Medical University, Xi'an 710038, China

**Keywords:** 胸壁重建, 材料, 3D打印, 聚醚醚酮, Chest wall reconstruction, Materials, 3D-printing, PEEK

## Abstract

肿瘤切除或外伤等多种因素均有可能导致胸壁缺损，骨性胸壁缺损的重建依旧是胸外科手术的重点和难点。随着材料学的不断进展，越来越多的新材料运用于骨性胸壁重建，一定程度上推动了胸壁外科的发展。但是，目前临床上常用的重建材料尚无一种能完全满足临床工作的需要。随着计算机技术的高速发展，3D打印技术也逐步运用于临床，个性化治疗理念逐步深入人心。个性化治疗配合新材料的使用极有希望克服现有材料的不足，使胸壁外科达到一个新高度。因此，本文对缺损胸壁重建的发展和现状做一综述，并对其发展方向做一积极展望。

## Abstract (en)

Chest wall defect may be caused by many factors such as the resection of tumor and trauma, and the reconstruction of bone-defection is still the key point of thoracic surgery. With the development of material science, more and more new materials have been used in medical practice, which makes huge progress in the surgery of chest wall. However, none of these materials satisfy all the practical needs of the reconstruction. Recently, with the development of the capacity of computer, 3D-printing technology has been gradually used in clinical work, and the idea of individual treatment has been accepted by more and more people. The weakness of these materials may be solved by the new material and the application of individual treatment, which could also make great advance in chest wall surgery. This article will make a summary of the research on the reconstruction of chest wall.

胸壁的完整性和稳定性对保护胸腔和纵隔内重要脏器及维持正常呼吸功能发挥关键作用，各种原因导致的胸壁缺损会破坏胸廓完整性和稳定性，使患者出现反常呼吸、呼吸功能障碍等症状。如果胸壁疾病再合并胸腔内器官的病变或者创伤，将会大大地增加治疗难度。因此，根据患者具体情况，对胸壁大块缺损进行个体化治疗及重建十分重要。

## 1 胸壁重建的适应症及重建原则

胸壁重建需要外科医生具有极强的创新能力和应变能力，目前尚没有统一的胸壁重建指南。多数胸外科医师普遍认为：针对单纯的胸壁缺损，在不累及胸骨或脊柱的情况下，前胸缺损直径小于5 cm，后胸壁缺损小于10 cm，可考虑直接缝合周围组织^[1]^。但全层大块胸壁缺损，范围超过6 cm×6 cm且相连3根以上肋骨受损时，或者累及胸骨、脊柱等情况下应考虑及时完成胸壁骨性重建，以恢复胸廓的完整性和稳定性，否则将不可避免地出现胸壁软化和反常呼吸等情况，加重呼吸、循环紊乱^[2]^。

胸壁重建包括骨性重建和软组织重建两部分。前者是利用修补材料恢复胸壁的坚固性和稳定性，后者则借助转移组织瓣实现胸壁的密闭性和较好的外观。后者相对简单和成熟，最早Tansini等^[3]^在1906年就报道了背阔肌带蒂皮瓣修复前胸壁缺损的经验。而由于各种材料性质的限制，胸壁重建的难点和重点在于骨性重建。胸壁重建一般要考虑以下几个因素：①能够保护胸腔及上腹部的脏器；②保证呼吸功能的完整性；③支持上肢和肩关节动作完成和力量；④充分考虑美观，恢复伤者自信^[4, 5]^。一般认为，理想的胸壁修补材料应具备：①足够的坚硬度，能确保胸廓稳定和保护胸内重要组织、器官，防止反常呼吸；②可植入性，不致癌，允许纤维组织覆壁生长，同时不易感染；③具有可塑形，易于制作成形，贴合胸廓外形；④不影响胸部X线检查，对患者可进行随访^[6]^。目前国内外针对胸壁缺损的骨性重建已开展了多项尝试，并随着材料学和计算机等基础学科的发展取得了较大的进展，虽然各种方法均存在不同程度的缺陷，但是越来越多的患者受益于新技术和新策略的使用。在此，我们对目前现有的重建方案进行回顾和分析，并展望新材料的使用。

## 2 传统重建材料及使用

现有的临床病例报道中，重建胸壁缺损的材料主要分为以Peri-Guard、VERITAS Collagen Matrix、Permacol等为代表的生物性材料；以Vypro、Vicryl、Gore-Tex、Prolene、Premilene、Parietex Composite、Parietene等为代表的人工材料；以钛合金为代表的金属材料以及患者自身的骨性组织等。目前已经应用于修补的材料虽较多，但尚无一种材料能完全达到上述要求^[7, 8]^。

### 2.1 生物性材料

Wiegmann和Makarawo等^[7, 9]^比较了生物学材料的特性，证实：生物性材料的毒性小，对细胞的活性基本没有影响，因此可考虑作为细胞贴附的基底面。同时也提出虽然生物性材料的显示出更好的细胞覆壁能力，但是由于其表面不够光滑，他们同样具有良好的细菌贴附能力，抗感染能力较差。因此，目前认为如果在无菌程度更高的部位使用生物性材料会更合适。

### 2.2 人工合成材料

Huang和Nazerali等^[10, 11]^分别使用合成材料进行胸壁重建，并比较合成材料的特点。合成材料中除了Gore-Tex外，其他材料的生物组织相容性均劣于生物性材料，易出现排异反应，可合并严重的并发症。胸壁重建后需要用肌肉覆盖置入物质，因此胸壁重建更倾向选择组织相容性好的材料，利于细胞贴附。Gore-Tex主要由碳和氟元素合成，具有很好的组织相容性，是一种多微孔材料，能够被纤维组织覆盖并穿透；另外，此材料对水和空气不具有渗透性。

无论是生物性材料还是合成材料，目前应用于临床的主要是软的可塑性网状或片状合成材料，他们不能提供完整胸廓所具有的稳定性和完整性以妥善保护胸内脏器、维护正常的呼吸和循环功能。为了保证重建胸壁的稳定性，Hummelink和Rocco等^[12, 13]^分别使用双层生物材料内夹骨水泥，以" 三明治" 式修复保证胸壁的稳定性。但是，此种方法由于重建材料中骨水泥的弹性差、射线穿透性差、组织相容性不佳等缺点只能用于相对局限的、平坦的胸壁部分，不能用于大多数胸壁重建。

### 2.3 金属材料

金属材料主要包括金属丝、网、板。由于金属材料良好的力学性能和可加工性，对胸壁支撑作用好，一直是胸壁骨性缺损修复的可选材料之一。但其弊端也很明显，感染、固定、出血、疼痛、穿破胸内脏器等危险经常发生，使得大多数金属材料的应用逐渐减少。钛及钛合金具有无毒、质轻、良好的生物相容性、机械强度和耐腐蚀性能以及对X线检查影响较小等特性，是理想的医用金属材料^[14]^。但是，由于胸壁缺损的类型和大小各不相同，统一生产的钛合金材料不能满足胸壁重建对重建形态的要求^[15]^。

### 2.4 自体骨性组织

自体骨性组织具有良好的生物相容性、较好的坚韧性和抗感染抗排斥作用，自体骨移植能立即恢复胸壁的骨性结构而不出现生物耐受性的问题，是最佳的重建材料，但是，自体骨移植会增加手术创伤、取材有限、供体区疼痛等不足，限制了自体肋骨的应用^[16]^。

## 3 新技术和新材料的探索性使用

### 3.1 3D打印技术运用于胸壁重建

产业化生产的产品中，能对胸壁进行支撑的材料一般都具有相对固定的形状，不能完整贴合胸壁外形，胸壁修复不完整、美观作用较差。近年来，随着个体化治疗的逐步开展和计算机技术的飞速提高，3D打印技术开始运用于胸腔外科手术，尤其是胸壁重建手术，以患者自身胸壁形状作为打印模板的3D打印肋骨、胸骨已逐渐用于临床。

3D打印技术能够在短时间内利用计算机成像技术制作出1:1的实物。从胸壁重建的角度来看，患者的解剖和需求不尽相同，批量生产的器械耗材具有完全相同的形状的尺寸，不能满足每个患者的需要。而3D技术为每个患者进行量体裁衣，制定个体化治疗方案，能够使患者获得最大的益处。

3D打印，即快速成型技术的一种，出现在20世纪90年代中期，它是一种以数字模型文件为基础，利用光固化和纸层叠等技术的最新快速成型装置。它与普通打印工作原理基本相同，打印机内装有液体或粉末等" 打印材料" ，与电脑连接后，运用粉末状金属或塑料等可粘合材料，通过电脑控制把" 打印材料" 一层层叠加起来，最终把计算机上的蓝图变成实物。因此，3D打印技术能够有效发挥各种材料的优越性。

### 3.2 3D打印材料的优化

目前，使用得最多的3D打印重建材料以钛合金报道最多，2015我国学者年和西班牙学者分别利用3D打印钛合金完成胸骨重建，尤其是我国学者最大限度保持了患者术前正常的胸廓解剖形态，完整恢复患者胸廓，显著改善患者术后心肺功能，提高患者生活质量^[17]^。此后，我科继续稳步推进3D打印在胸壁重建的运用，经过数次改进，利用3D打印钛合金完成多个肋骨及胸肋骨联合1:1的重建的工作，并有效减轻了重建骨性结构的重量^[18]^。西班牙团队则更强调优化打印功能细节，例如，加固胸肋骨的连接处、打印弹性钛合金及可伸缩结构，以提高3D打印的个性化制作优势^[19, 20]^。3D打印技术运用于钛合金能够有效兼顾重建材料的形态匹配性及其组织相容性、抗感染等能力等优势，更符合手术要求。

3D打印钛合金虽然具有上述优势，但是同样具有一定的局限性，主要集中在：①钛合金与皮质骨弹性模量和屈曲强度差异过大，易导致其他部位损伤；②大范围修复引起的限制性肺通气障碍；③影像学干扰等。而聚醚醚酮（poly-ether-ether-ketone, PEEK），具有耐高温、耐化学腐蚀、耐辐射、生物相容性良好、计算机断层扫描（computed tomography, CT）-磁共振无干扰以及相对便宜的价格优势，能够较好地弥补这些不足。

PEEK是英国ICI公司于1977年开发成功，在20世纪80年代初期由英国Victrex公司实现工业化的一种高性能特种工程塑料。PEEK主要是以4, 4' -二氟二苯甲酮、对苯二酚、无水碳酸钠为原料，二苯砜为溶剂，在无水条件下于300 oC-340 oC进行亲核缩聚合制得。由于PEEK的大分子链结构规整，且含有刚性的苯环、柔性的醚键及可促进分子间作用力的羰基。因此，具有耐热等级高、耐辐射、耐化学药品腐蚀、耐蠕变，抗冲击性能、抗疲劳性能好等特点^[21]^，在许多特殊领域可以替代金属、陶瓷等传统材料，成为当今最热门的高性能特种工程塑料之一，在航空航天、电子电气、化工与机械工业、汽车制造业、食品加工业等领域得到广泛应用，并成为不可或缺的关键材料。PEEK及其复合材料具有良好的生物相容性，得到了美国食品药品监督管理局（Food and Drug Administration, FDA）的认可；PEEK还具有生物惰性，根据ISO 10993-10:1995，证明其不具有致敏性，基因毒性测试结果显示其不会引起任何染色体畸变。因此，PEEK及其复合材料在生物医用领域应用前景广阔^[22]^。

近年来，张珏^[23]^利用熔融沉积成型原理进行PEEK仿生人体内踝的3D打印制造，打印前后PEEK未发生明显化学变化且与细胞相容性较好。提示PEEK-3D打印应用于临床已具有理论基础。由于3D打印技术能够根据不同患者需要，制备适合不同患者的个性化生物医用材料，同时可精确控制材料的微观结构，凭借PEEK-3D打印的这种独特优势，其将在未来的生物医学领域占据重要地位^[24]^。我科室目前已成功利用3D打印PEEK材料完成了胸骨、肋骨、胸肋骨联合重建等临床工作，患者均顺利恢复，术后重建胸廓外形较术前无改变，由于大大降低了重建材料的重量，患者术后无任何异物、不适感，术后满意度极高。

### 3.3 组织工程骨修复

除此之外，不少学者还在利用组织工程技术制作新型重建材料。此部分工作目前还处于动物实验阶段，尚未推向临床。该方法有以下几个特点：①利用患者自提的MSC诱导分化成骨细胞；②利用3D打印技术制作生物支架；③将分化的成骨细胞种植于3D打印支架上，利用组织工程技术培养人工骨。该技术弥补了现有3D打印技术组织相容性的弱项，能提供更符合人体需要的重建材料。

总之，胸壁重建作为胸外科治疗的一大难题已随着材料学和计算机等发展取得了巨大的进步，虽然目前各种材料均存在部分缺陷，但是随着各种新材料、新技术地不断涌出，胸壁缺损将得到越来越妥善的重建和修复。
